# Assessment of cardiac function in rat endovascular perforation model of subarachnoid hemorrhage; A model of subarachnoid hemorrhage-induced cardiac dysfunction

**DOI:** 10.3389/fnsyn.2022.919998

**Published:** 2022-08-09

**Authors:** Masahito Munakata, Hideaki Kanazawa, Kensuke Kimura, Takahide Arai, Hiroaki Sukegawa, Keiichi Fukuda

**Affiliations:** ^1^Department of Cardiology, Saitama City Hospital, Saitama, Japan; ^2^Department of Cardiology, Keio University School of Medicine, Tokyo, Japan; ^3^Kimura Internal Clinic, Kanagawa, Japan; ^4^Division of Cardiology, Tokyo Dental College Ichikawa General Hospital, Chiba, Japan

**Keywords:** subarachnoid hemorrhage, animal model, cardiac dysfunction, arrhythmia, CXCL-1 chemokine, CCL-2, sympathetic nerve, catecholamine

## Abstract

Although the association between cardiac dysfunction and subarachnoid hemorrhage (SAH) has been recognized, its precise underlying mechanism remains unknown. Furthermore, no suitable animal models are available to study this association. Here, we established an appropriate animal model of SAH-induced cardiac dysfunction and elucidated its mechanism. In this rat model, contrast-enhanced computed tomography of the brain confirmed successful induction of SAH. Electrocardiography detected abnormalities in 55% of the experimental animals, while echocardiography indicated cardiac dysfunction in 30% of them. Further evaluation of left ventriculography confirmed cardiac dysfunction, which was transient and recovered over time. Additionally, in this SAH model, the expression of the acute phase reaction protein, proto-oncogene c-Fos increased in the paraventricular hypothalamic nucleus (PVN), the sympathetic nerve center of the brain. Polymerase chain reaction analysis revealed that the SAH model with cardiac dysfunction had higher levels of the macrophage-associated chemokine (C-X-C motif) ligand 1 (CXCL-1) and chemokine (C-C motif) ligand 2 (CCL-2) than the SAH model without cardiac dysfunction. Our results suggested that SAH caused inflammation and macrophage activation in the PVN, leading to sympathetic hyperexcitability that might cause cardiac dysfunction directly and indirectly. This animal model may represent a powerful tool to investigate the mechanisms of the brain-heart pathway.

## Introduction

Acute subarachnoid hemorrhage (SAH) is a serious disease, affecting both brain and other organs, such as the heart. The estimated frequency of SAH is 6.9–9 per 100,000 persons per year, with ruptured cerebral aneurysms as the most common cause. Currently, SAH is a fatal disease with a 30-day mortality rate of up to 45% (Osgood, 2021). Coiling and clipping are used to treat SAH; however, the return-to-society rate is as low as 30%. Therefore, treatment of SAH-associated comorbidities is also important to improve prognosis. SAH-associated cardiac abnormality is a well-known phenomenon to neurosurgeons and cardiologists, which was first documented in the 1950s (Shuster, 1960). Many studies reported a correlation between electrocardiography (ECG) and transthoracic echocardiography (TTE) abnormalities with SAH (Macrea et al., 2005). In this regard, a large number of ECG abnormalities are transient (Kono et al., 1994); however, they can sometimes cause life-threatening arrhythmias, such as ventricular tachycardia, ventricular fibrillation, and Torsade de Pointes (Lanzino et al., 1994). Additionally, QT prolongation has been reported as a major SAH-associated abnormality (Komatsuzaki et al., 2021). In terms of TTE abnormalities, a previous study demonstrated that the more severe the SAH, the more likely is the occurrence of TTE changes (Macrea et al., 2005). Although over 70 years have elapsed since the initial report of SAH-associated cardiac abnormality, the mechanism of this phenomenon remains unknown and a suitable animal model has not yet been established. In the human body, biological functions are maintained by the close coordination of multiple organs, not by a single organ. In this regard, the brain and the heart are thought to be closely linked. However, the precise relationship between these two organs is not yet fully understood.

## Materials and methods

### Animal preparation

Wistar rats (250–300 *g*, male, 10–12 weeks old; Nihon CLEA, Tokyo, Japan) were used in this study. Animals were maintained under controlled temperature and allowed free access to food and water before and after the study procedure. The rats were anesthetized with an intraperitoneal injection of sodium pentobarbital [pentobarbital Na (30–40 mg/kg) + butorphanol tartrate (2.5 mg/kg)]. This study conformed to the Guide for the Care and Use of Laboratory Animals published by the United States National Institutes of Health (NIH publication no. 85-23, revised 1996) and was approved by the Institutional Animal Care and Use Committee at the Keio University School of Medicine.

### Surgical procedure

**FIGURE 1:**
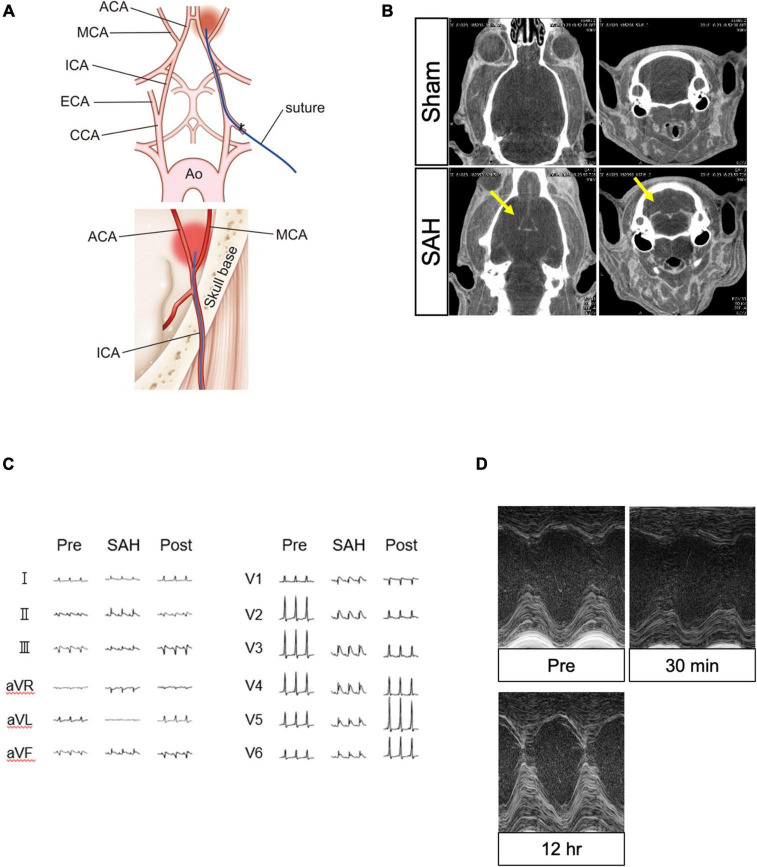
Surgical procedure for creating the SAH model and confirmation of the model using several modalities. **(A)** Surgical procedure to create the model of SAH puncture. **(B)** Contrast leakage was visible in the subarachnoid space in the SAH group (yellow arrow) but not in the sham group. **(C)** ECG changes in the SAH group recorded before, during, and after the SAH procedure. **(D)** TTE changes in the SAH group obtained before and after the SAH procedure. ACA, anterior cerebral artery; Ao, aorta; CCA, common carotid artery; ECA, external carotid artery; ECG, electrocardiography; ICA, internal carotid artery; MCA, middle cerebral artery; SAH, subarachnoid hemorrhage; and TTE, echocardiography.

The SAH model of vascular perforation in rats was previously established (Bederson et al., 1995). The rats were placed in the supine position on a heated table. Using an operating microscope, the left carotid artery was exteriorized through a skin incision at the neck. The external carotid artery (ECA) was ligated and cut down near the internal carotid artery (ICA) bifurcation. A 4-0 nylon suture with a sharpened tip was inserted approximately 20 mm toward the skull base through the ICA from the ECA edge. The suture tip accurately pierced through the bifurcation of the anterior and middle cerebral arteries, resulting in SAH (Figure 1A). SAH induction was confirmed at the time of analysis. Animals in the sham group underwent the same operation but without the vessel puncture.

### Head computed tomography

To confirm the success of the SAH procedure, head computed tomography (CT) scanning was performed immediately after SAH was induced. After inducing anesthesia, a 3-mL contrast medium (Iopamiron, Bayer Yakuhin, Ltd, Osaka, Japan) was injected through the exteriorized left femoral vein. Bolus injection of contrast medium was manually provided before SAH induction. Next, we conducted the SAH procedure and CT scanning (R_mCT2, Rigaku, Tokyo, Japan). Head CT was similarly performed in both the sham and SAH groups.

### Heart rate, systolic blood pressure, electrocardiography, and transthoracic echocardiography

Heart rate (HR) and blood pressure (BP) were assessed before and after the SAH procedure by the tail-cuff method (BP-98A-L, Softron, Tokyo, Japan). BP and HR were performed before and 30 min after SAH. The measurements were performed three times for each animal and the average value was adopted. A 12-lead ECG was performed using a scanner (ECG-1350, Nihon Koden, Tokyo, Japan) with microneedle electrodes, while TTE was sequentially recorded before and after the SAH procedure (Vevo 770, Fujifilm Visual Sonics Inc., Toronto, Canada) with 17.5- and 30-MHz probes. In most animals, TTE and ECG were performed before and at 30 min, 60 min, 90 min, 120 min, 12 h, and 24 h after SAH operation. Left ventricular systolic diameter and left ventricular diastolic diameter were measured in the parasternal short axis, from which ejection fraction (EF) was calculated by the Teichholz method. Since no local asynergy was observed, EF could be computed by this method. EF was measured by a blinded evaluator, with a 21-MHz frame rate and 400-bpm HR. Inhaled anesthesia was used for TTE (1–2% inhaled isoflurane).

### Coronary artery angiography

The rats were anesthetized, and a 0.58-mm polyethylene tube was inserted into the right common carotid artery and guided into the ascending aorta. Contrast medium was injected *via* the tube and synchrotron radiation angiography was performed (HITEX, Osaka, Japan). Synchrotron radiation was derived from an electron beam of a 6.5-GeV storage ring using a bending magnet. The synchrotron radiation beam has a broad wavelength and is monochromatized and magnified by reflecting it off an asymmetrically cut silicon crystal placed in front of the subject. The final photon density of the monochromatized X-ray used in our experiment was estimated to be approximately 10,000-fold higher than those of conventional X-ray sources. Therefore, synchrotron radiation microangiography provided detailed and high-contrast X-ray images. Coronary angiograms were obtained in both SAH and sham groups. Coronary artery angiography (CAG) were imaged approximately 30 min after SAH preparation.

### Left ventriculography

A 0.58-mm polyethylene tube was inserted from the internal cervical artery into the left ventricle of anesthetized rats. Tube placement in the left ventricle was confirmed by evaluating the pressure waveforms. From the tube, 1-mL contrast medium was injected and left ventriculography (LVG) of the SAH and sham groups was performed (HITEX, Osaka, Japan). LVG were imaged approximately 30 min after SAH preparation.

### Cerebrospinal fluid, blood, and brain analyses

After the various measurements, the animals were sacrificed under deep anesthesia with pentobarbital sodium (50–100 mg/kg, administered intraperitoneally). The anesthetized rats were placed in a stereotaxic frame (SR-6R, Narishige, Tokyo, Japan), and a small incision was made between the neck and head. Tissue was decorticated with forceps and tweezers, and the atlanto-occipital membrane was exposed. Cerebrospinal fluid (CSF) was aspirated through the atlanto-occipital membrane using a 29G needle, and interleukin (IL)-6 and IL-1β levels of the CSF were analyzed. IL-6 and IL-1β were measured by the chemiluminescence enzyme immunoassay and enzyme-linked immunosorbent assay methods, respectively, (SRL, Tokyo, Japan). Additionally, a blood sample was obtained from the inferior vena cava.

The severity of SAH was evaluated by grading the hemorrhagic area of the cerebral base according to an established scale (Sugawara et al., 2008). The basal cistern was divided into six segments. Each segment was given a grade from 0 to 3 depending on the amount of subarachnoid blood clot as follows: Grade 0, no subarachnoid blood; Grade 1, minimal subarachnoid blood; Grade 2, moderate blood clot with recognizable arteries; and Grade 3, blood clot obliterating all the arteries within the segment. The animals received a total score ranging from 0 to 18 after adding the scores of all six segments. According to the final score, the animals were divided into three groups as follows: 0–7, mild SAH; 8–12, moderate SAH; and 13–18, severe SAH.

### Polymerase chain reaction analysis

mRNA extraction and quantitative reverse-transcription polymerase chain reaction (PCR) were performed as previously described (Ieda et al., 2004) using the ABI Prism 7500 Sequence Detection System (Applied Biosystems). All samples were analyzed in triplicates. The primers and TaqMan probe for CCl-2 (Rn00580555_m1), CXCL-1 (Rn00578225_m1), and c-Fos (Rn02396759_m1) were purchased from Applied Biosystems. All mRNA levels were normalized against those of GAPDH. The paraventricular hypothalamic nucleus (PVN), the sympathetic nerve center of the brain, was sampled and analyzed to detect changes in mRNA expression in the SAH and sham groups.

### Immunofluorescence staining

Brains of the experimental rats were perfused *via* the inferior vena cava with phosphate-buffered saline, followed by 4% paraformaldehyde. After perfusion, the brains were removed, fixed overnight, and cryoprotected in 30% sucrose at 4°C for 7 days before being embedded in optimal cutting temperature compound and then frozen in liquid nitrogen. Cryostat sections (7 μm) were stained with antibodies against c-Fos (sc-52; Santa Cruz Biotechnology, Santa Cruz, CA, United States) to detect nerve activity. Sections were then incubated with secondary antibodies conjugated with Alexa Fluor 488 dye and nuclei were counterstained with 4′,6-diamidino-2-phenylindole. Confocal microscopy was performed on an LSM 510 META Confocal Microscope (Carl Zeiss, Oberchen, Germany).

### Statistical analysis

Data were presented as mean ± standard deviation. Statistical comparisons between two groups were determined by two-tailed, unpaired Student’s *t*-tests, while those of multiple groups were determined by one-way ANOVA and *post hoc t*-tests. *P* < 0.05 was considered statistically significant. Statistical analyses were performed using IBM SPSS Statistics, Version 21.0 (SPSS, Inc., Chicago, IL, United States).

## Results

### Head computed tomography

Computed tomography images were acquired in the axial and coronal views. The SAH group showed a clear flow of contrast medium into the subarachnoid space of the brain in comparison to the sham group, confirming the induction of SAH (Figure 1B).

### Electrocardiography

At 2 h after the SAH procedure, 55% of the animals in the SAH group (*N* = 31) showed ST-segment elevation in the precordial and limb leads on ECG. The change was observed in the wide range leads, but without accompanying abnormal Q wave and ST segment depression. Additionally, no life-threatening arrhythmia, such as ventricular tachycardia/ventricular fibrillation, was detected. The SAH-induced ECG changes were transient and normalized after 24 h (Figure 1C).

### Transthoracic echocardiography

**FIGURE 2:**
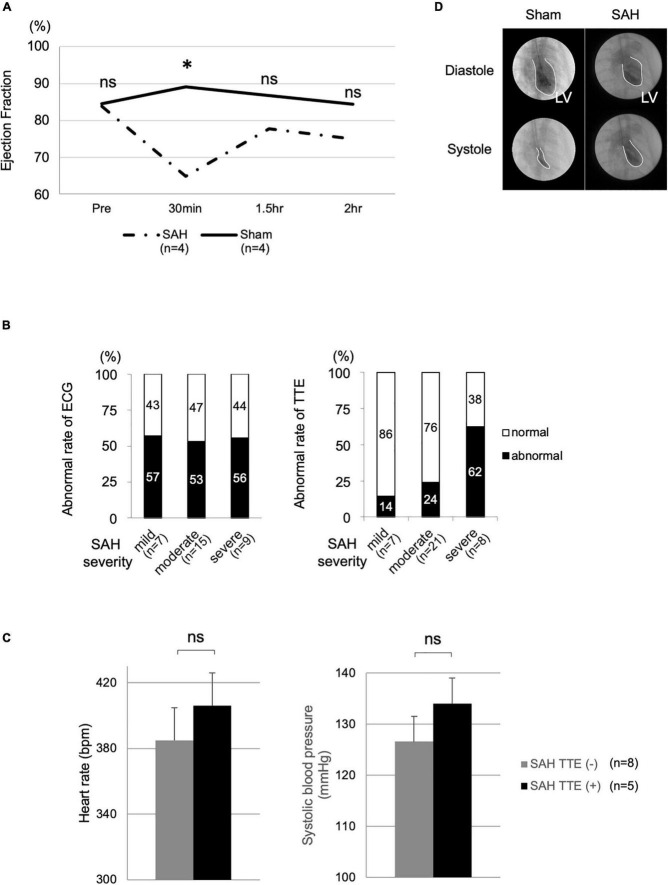
Cardiac dysfunction and vital signs in the SAH and sham groups. **(A)** TTE measurements of the SAH (*N* = 4) and sham (*N* = 4) groups at various time points. **(B)** Correlations of the rates of ECG (mild, *N* = 7; moderate, *N* = 15; and severe, *N* = 9) and TTE changes (mild, *N* = 7; moderate, *N* = 21; and severe, *N* = 8) with SAH severity. **(C)** Heart rate and systolic blood pressure in the SAH group with TTE change [SAH TTE (+), *N* = 5] and the SAH group without TTE change [SAH TTE (−), *N* = 8]. **(D)** Cardiac function analysis using LVG. Bpm, beats per minute; ECG, electrocardiography; LV, left ventricle; LVG, left ventriculography; ns, not significant; SAH, subarachnoid hemorrhage; and TTE, echocardiography. *Indicated *P* < 0.05.

Left ventricular systolic function was evaluated using M-mode TTE. The results showed that 30% of the animals in the SAH group (*N* = 36) had a significant reduction in left ventricular contractility, which peaked at 30 min to 1 h after the SAH procedure. Similar to the ECG changes, SAH-induced TTE abnormalities were transient and normalized after few hours (Figures 1D, 2A).

### The rate of abnormalities and subarachnoid hemorrhage severity

The TTE abnormality rate was proportional to the severity of SAH (Figure 2B). In the TTE analysis, the mild, moderate, and severe groups included 7, 21, and 8 rats, respectively. The mortality rate of the SAH model was approximately 20%.

### Heart rate and blood pressure

Heart rate (*P* = 0.240) and BP (*P* = 0.672) were not significantly different between the SAH group without TTE change [SAH TTE (−), *N* = 8, mean HR after SAH: 384.9 ± 39.1 beats/min, mean BP: 126.6 ± 26.0 mm Hg] and the SAH group with TTE change [SAH TTE (+), *N* = 5, mean HR after SAH: 406.1 ± 19.6 beats/min, mean BP: 134 ± 27.8 mm Hg; Figure 2C].

### Coronary artery angiography

The coronary arteries of the SAH and sham groups did not show any occlusion, stenosis, or spastic lesion, indicating an almost normal coronary artery (Supplementary Figure).

### Left ventriculography

Left ventricular contractility was maintained normally in the sham group, whereas left ventricular contraction was significantly decreased in the SAH group. This change showed diffuse, but not focal, hypokinesis (Figure 2D).

### Cerebrospinal fluid and blood analyses

**FIGURE 3:**
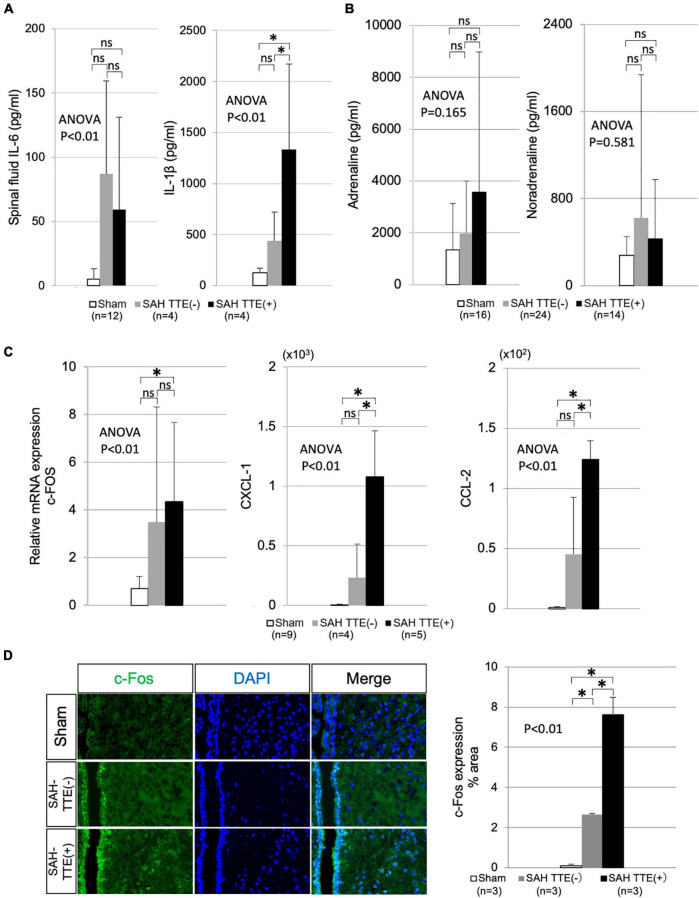
CSF, blood, and brain analyses in the SAH and sham groups. **(A)** Concentration of IL-6 and IL-1β in the CSF of the sham (*N* = 12), SAH TTE (−; *N* = 4), and SAH TTE (+; *N* = 4) groups. **(B)** Blood adrenaline and noradrenaline levels of the sham (*N* = 16), SAH TTE (−; *N* = 24), and SAH TTE (+; *N* = 14) groups. **(C)** mRNA expression of c-Fos, CXCL-1, and CCL-2 in the PVN of the sham (*N* = 9), SAH TTE (−; *N* = 4), and SAH TTE (+; *N* = 5) groups. **(D)** Immunostaining for c-Fos near the PVN and third brain ventricle in the sham (*N* = 3), SAH TTE (−; *N* = 3), and SAH TTE (+; *N* = 3) groups. CCL-2, chemokine (C-C motif) ligand 2; CXCL-1, chemokine (C-X-C motif) ligand 1 (CXCL-1); CSF, cerebrospinal fluid; IL, interleukin; PVN, paraventricular hypothalamic nucleus; and SAH, subarachnoid hemorrhage. *Indicated *P* < 0.05.

As shown in Figure 3A, IL-6 level showed no significant difference between the SAH TTE (−) and SAH TTE (+) groups, while IL-1β level showed a significant difference between two groups. Serum catecholamines, including adrenaline and noradrenaline, tended to be higher in the SAH group than in the sham group (Figure 3B).

### Brain analysis

Polymerase chain reaction analysis revealed that the mRNA expression levels of c-FOS in the PVN did not differ between the SAH TTE (−) and SAH TTE (+) groups. However, CXCL-1 and CCL-2 levels showed significant differences between two groups. Immunofluorescence staining of the PVN confirmed a significantly elevated expression of c-Fos in the SAH TTE (+) group than those of the sham or SAH TTE (−) groups (Figures 3C,D).

## Discussion

Subarachnoid hemorrhage is sometimes associated with cardiac complications. Clinically significant arrhythmias after SAH were associated with a high mortality rate and serious cardiac and neurological comorbidity (Jeon et al., 2010). Additionally, certain cardiac contractile disorders have been reported to be caused by the overlap of SAH with Takotsubo cardiomyopathy, which can be triggered by mental and physical stress (Ako et al., 2003). To date, a well-established animal model to investigate this association is not available. In this study, we successfully developed a new animal model of SAH-induced cardiac dysfunction. Using this model, we were able to acquire a clear CT image of SAH for the first time. The overall mortality rate of human SAH was reportedly 10–67% (Taylor et al., 1991; Neil-Dwyer et al., 1998; van Gijn and Rinkel, 2001), which was similar to the mortality rate of our model (20%).

The examination of this new model of SAH-induced cardiac dysfunction revealed reversible ECG and TTE changes, as observed in human SAH. Furthermore, TTE changes were particularly closely associated with the severity of SAH (Macrea et al., 2005), which was also demonstrated in our model. We confirmed SAH-associated cardiac wall motion abnormalities on both TTE and LVG. Although coronary artery spasm was the speculated cause of cardiac dysfunction in SAH (Chen, 2013), we affirmed that there was no obvious spasm on CAG in our SAH model. To date, there have been no reports of LVG or CAG in the rat models of SAH.

Since HR and BP were not different between the SAH TTE (−) and SAH TTE (+) groups, SAH, rather than the influence of vital signs, might have caused the cardiac dysfunction. Considering these results, we might have successfully created an appropriate animal model that mimics human SAH.

Previous studies have reported increased inflammatory CSF cytokines and increased serum catecholamines in animal models as well as in humans (Naredi et al., 2000; Fassbender et al., 2001; Kwon and Jeon, 2001; Masuda et al., 2002; Kawahara et al., 2003; Simon and Grote, 2021). In our model, CSF analysis showed increased levels of inflammatory cytokines, such as IL-1β and IL-6, while serum catecholamine levels among experimental groups. We presumed that SAH could have caused these increased levels *via* the activation of the sympathetic nerves and the induction of inflammation.

In the PVN, PCR analysis indicated enhanced mRNA expression of c-Fos, CXCL-1, and CCL-2, while immunostaining confirmed an increase in c-FOS at the protein level. C-Fos is a type of acute phase reaction protein, which is also used as a marker to assess the functional activity of the brain (Sheng and Greenberg, 1990; Martinez et al., 2002). The enhanced expression of c-Fos in the PVN around the brain ventricles might have resulted from increased functional activity of the site due to SAH. Moreover, CXCL-1 and CCL-2 (also known as monocyte chemoattractant protein 1) are associated with inflammation and macrophages (Atri et al., 2018). Lu et al. (2009) reported increased CCL-2 expression in the cerebral artery of a rat model of SAH. This elevated expression of chemokines suggested that SAH might activate inflammation and macrophages, resulting in macrophage migration to the vicinity of the PVN.

Considering these results, we surmised that macrophage migration to the PVN was induced by SAH, leading to the secretion of inflammatory cytokines. Consequently, the sympathetic nerves were activated, resulting in the secretion of endogenous catecholamines to maintain homeostasis. Although sympathetic activation of catecholamine secretion helps maintain homeostasis, excess catecholamines can sometimes have harmful effects on the body. Excess catecholamines exert stress on the cardiomyocytes directly and indirectly, causing cardiac dysfunction. In support of this theory, patients with pheochromocytoma, which also involves the activation of the sympathetic nerves, have been reported to develop a cardiac dysfunction called catecholamine cardiomyopathy (Kassim et al., 2008). Nevertheless, the precise molecular mechanisms of this phenomenon have not yet been clarified and require further elucidation. Therefore, our model might represent a powerful tool to investigate the mechanisms of this brain-heart pathway.

## Conclusion

We successfully assessed animal model of SAH with cardiac dysfunction that mimics human SAH in various aspects. This study suggested that sympathetic overactivation was associated with cardiac dysfunction. However, the analysis of the pathway connecting the brain and heart and the heart itself remains incomplete and needs to be further investigated in the future.

## Data availability statement

The original contributions presented in this study are included in the article/Supplementary material, further inquiries can be directed to the corresponding authors.

## Ethics statement

The animal study was reviewed and approved by Institutional Animal Care and Use Committee at the Keio University School of Medicine.

## Author contributions

MM, HK, KK, and KF designed the study. MM, TA, and HS performed all animal procedures. MM, HK, and KK analyzed all data and provided administrative assistance. The manuscript was written by MM, HK, and KF. All authors contributed to the article and approved the submitted version.

## Supplementary material

Supplementary Figure Coronary artery angiography in the sham and SAH model. SAH, subarachnoid hemorrhage.
