# Supplementary figures and images for: Assessment of cardiac function in rat endovascular perforation model of subarachnoid hemorrhage; A model of subarachnoid hemorrhage-induced cardiac dysfunction
Source: Front Synaptic Neurosci. 2022 Aug 9;14:919998. doi: 10.3389/fnsyn.2022.919998 (PMC9396209; doi:10.3389/fnsyn.2022.919998)

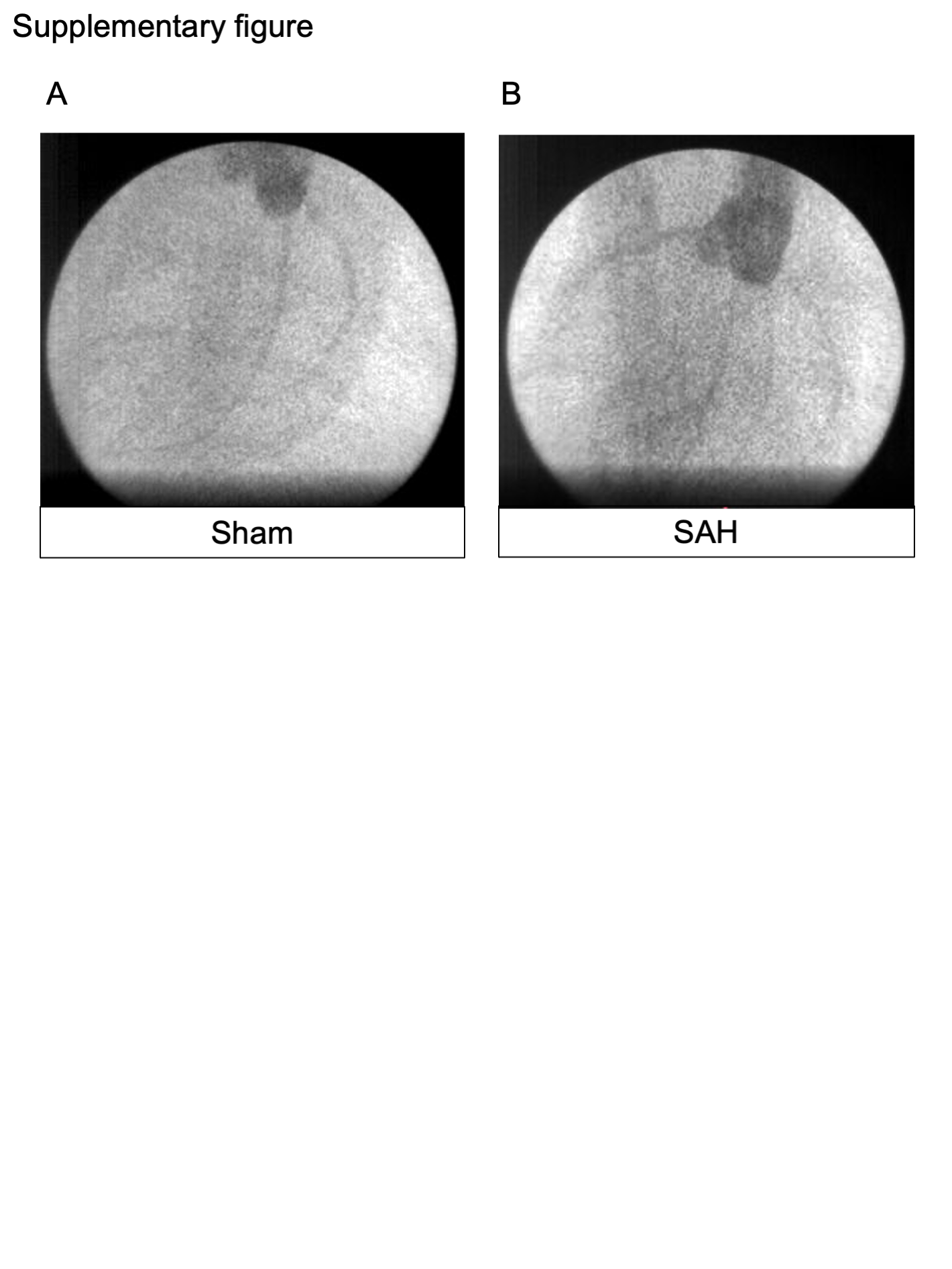

Supplement: Supplementary Figure — Coronary artery angiography in the sham and SAH model. SAH, subarachnoid hemorrhage. [file Image_1.tiff]
